# High Myc expression and transcription activity underlies intra-tumoral heterogeneity in triple-negative breast cancer

**DOI:** 10.18632/oncotarget.15891

**Published:** 2017-03-03

**Authors:** Nidhi Gupta, Karen Jung, Chengsheng Wu, Abdulraheem Alshareef, Hind Alqahtani, Sambasivarao Damaraju, John R. Mackey, Sunita Ghosh, Siham Sabri, Bassam S. Abdulkarim, Gilbert Bigras, Raymond Lai

**Affiliations:** ^1^ Department of Laboratory Medicine and Pathology, University of Alberta, Edmonton, Alberta, Canada; ^2^ Department of Oncology, University of Alberta, Edmonton, Alberta, Canada; ^3^ Cross Cancer Institute, Alberta Heath Services, Edmonton, Alberta, Canada; ^4^ Department of Oncology, McGill University, Montreal, Quebec, Canada; ^5^ DynaLIFE_Dx_ Medical Laboratories, Edmonton, Alberta, Canada

**Keywords:** triple-negative breast cancer, Myc, transcription activity, intra-tumoral heterogeneity, MAPK/ERK pathway

## Abstract

We have previously identified a novel intra-tumoral dichotomy in triple-negative breast cancer (TNBC) based on the differential responsiveness to a reporter containing the Sox2 regulatory region-2 (SRR2), with reporter responsive (RR) cells being more stem-like than reporter unresponsive (RU) cells. Using bioinformatics, we profiled the protein-DNA binding motifs of SRR2 and identified Myc as one of the potential transcription factors driving SRR2 activity. In support of its role, Myc was found to be highly expressed in RR cells as compared to RU cells. Enforced expression of *MYC* in RU cells resulted in a significant increase in SRR2 activity, Myc-DNA binding, proportion of cellsexpressing CD44^+^/CD24^–^, chemoresistance and mammosphere formation. Knockdown of Myc using siRNA in RR cells led to the opposite effects. We also found evidence that the relatively high ERK activation in RR cells contributes to their high expression of Myc and stem-like features. Using confocal microscopy and patient samples, we found a co-localization between Myc and CD44 in the same cell population. Lastly, a high proportion of Myc-positive cells in tumors significantly correlated with a short patient survival. In conclusion, inhibition of the MAPK/ERK/Myc axis may be an effective approach in eliminating stem-like cells in TNBC.

## INTRODUCTION

Triple-negative breast cancers (TNBC) are defined as tumors that lack the expression of estrogen receptor (ER), progesterone receptor (PR), and human epidermal growth factor receptor 2 (HER2) [1]. TNBC is the subtype of breast cancer that carries the worst clinical outcome [2]. Accumulating evidence suggests that TNBC is a highly heterogeneous disease at the genetic level [3]. Similar to many others solid tumors, TNBC comprise a subset of cells carrying stem-like features, commonly referred to as cancer stem cells (CSCs) [4]. In these tumors, CSCs have been shown to contribute to metastasis [5], chemoresistance [6] and poor clinical outcomes [7]. Nonetheless, the molecular biology and protein drivers of cells carrying stem-like features in the context of intra-tumoral heterogeneity remains to be fully understood.

The Sox2 regulatory region 2 (SRR2) is a conserved enhancer for the *SOX2* gene [8]. Recently, we have published that the activation of a SRR2-regulated, dual green fluorescence protein (GFP) and luciferase reporter construct was a good marker for the identification of a novel, intra-tumoral, phenotypically-distinct cell population in TNBC cell lines and primary TNBC patient tumors [9]. Specifically, we identified a very small subset of cells that were reporter responsive (RR), detectable based on their expression of GFP and luciferase; in contrast, the majority of cells were reporter unresponsive (RU), and they do not express GFP or luciferase [9]. We purified RU and RR cells from TNBC cell lines for further characterization and found that RR cells exhibited a larger CD44^+^/CD24^−^ cell population as compared to their RU counterparts [9]. Moreover, these RR cells were more tumorigenic *in vivo* and formed more mammospheres and Matrigel colonies *in vitro* [9]. Previously, we also had demonstrated that the SRR2 reporter responsiveness was heterogeneous within estrogen receptor positive (ER+) breast cancer cell lines and patient tumors [10, 11]. Furthermore, RR cells derived from ER+ breast cancers also exhibited enhanced tumorigenic capacity *in vivo* and *in vitro* [10]. Intriguingly, unlike the ER+ breast cancer cell lines which have robust Sox2 expression [10], we discovered that TNBC cells showed little to no expression of Sox2, and Sox2 was not a driver of the SRR2 reporter response [9]. This leads us to elucidate further mechanisms driving the SRR2 reporter response and associated tumorigenic phenotype in TNBC cells.

The Mitogen Activated Protein Kinase (MAPK)/Extracellular signal-Regulated Kinase (ERK) pathway has been shown to regulate cancer stem-like features in TNBC [12, 13]. The MAPK pathway stabilizes downstream target Myc by phosphorylation at the serine 62 site and this has been demonstrated in ER+ breast cancer and other types of cancer [14–16]. Myc is an established oncoprotein [17, 18]. Higher expression of Myc and its downstream targets have been documented in breast cancer including TNBC [17, 18]. Further, Myc expression has been linked to normal and breast CSCs [18–20]. Importantly, Myc transcription activity and expression remain to be characterized in heterogeneous breast tumor cell sub-populations within a single tumor.

In this study, using our purified RU/RR cell sub-population study model, we have uncovered that the MAPK/ERK-regulated Myc pathway is higher in the RR cell sub-population as compared to the RU cell sub-population within TNBC cell lines. Furthermore, Myc is the key regulator of the observed tumorigenic and cancer stem-like features in RR cells within these TNBC cell lines. Myc is more transcriptionally active in RR cells. In primary TNBC patient tumors, we found that Myc significantly co-localized with CD44 in a subset of cells. We also found that a high proportion of cells expressing Myc in TNBC patient tumors significantly correlate with short overall survival. Taken together, using this RU/RR cell sub-population study model, we have gained insights into the differential Myc expression and transcription activity that underlie the biology of cancer stemness in TNBC.

## RESULTS

### Myc expression and activity are substantially different between RU and RR cells

We have previous ly published that Sox2 does not play a key role in contributing to the differential SRR2 reporter activity or tumorigenic properties in the purified RU/RR cells derived from TNBC cell lines [9]. In this study, we aimed to identify the alternative transcription factor(s) that might be responsible for the differential SRR2 reporter responsiveness in TNBC. To achieve this goal, we employed bioinformatics to profile the protein-DNA binding motifs in the 81-bp SRR2 sequence. Using an *in silico* approach and the JASPAR database, we found 16 transcription factors, including Sox2, that show a high probability of binding to the SRR2 consensus sequence with a *p-value* < 0.001 (Supplementary Figure 1A).

To shortlist the important transcription factor(s), we evaluated the relative abundance of the mRNA of the 16 targets using quantitative reverse transcriptase-PCR (qRT-PCR). We found that MYC was the highest expressed MDA-MB-231 cells (supplementary Figure1B). Importantly, RR cells derived from MDA-MB-231 cells expressed significantly more *MYC* mRNA as compared to RU cells (Supplementary Figure 1B). In keeping with our previous results, *SOX2* was minimally expressed. We repeated the same experiment using another TNBC cell line, SUM-149, and similar results were obtained (data not shown).

**Figure 1:**
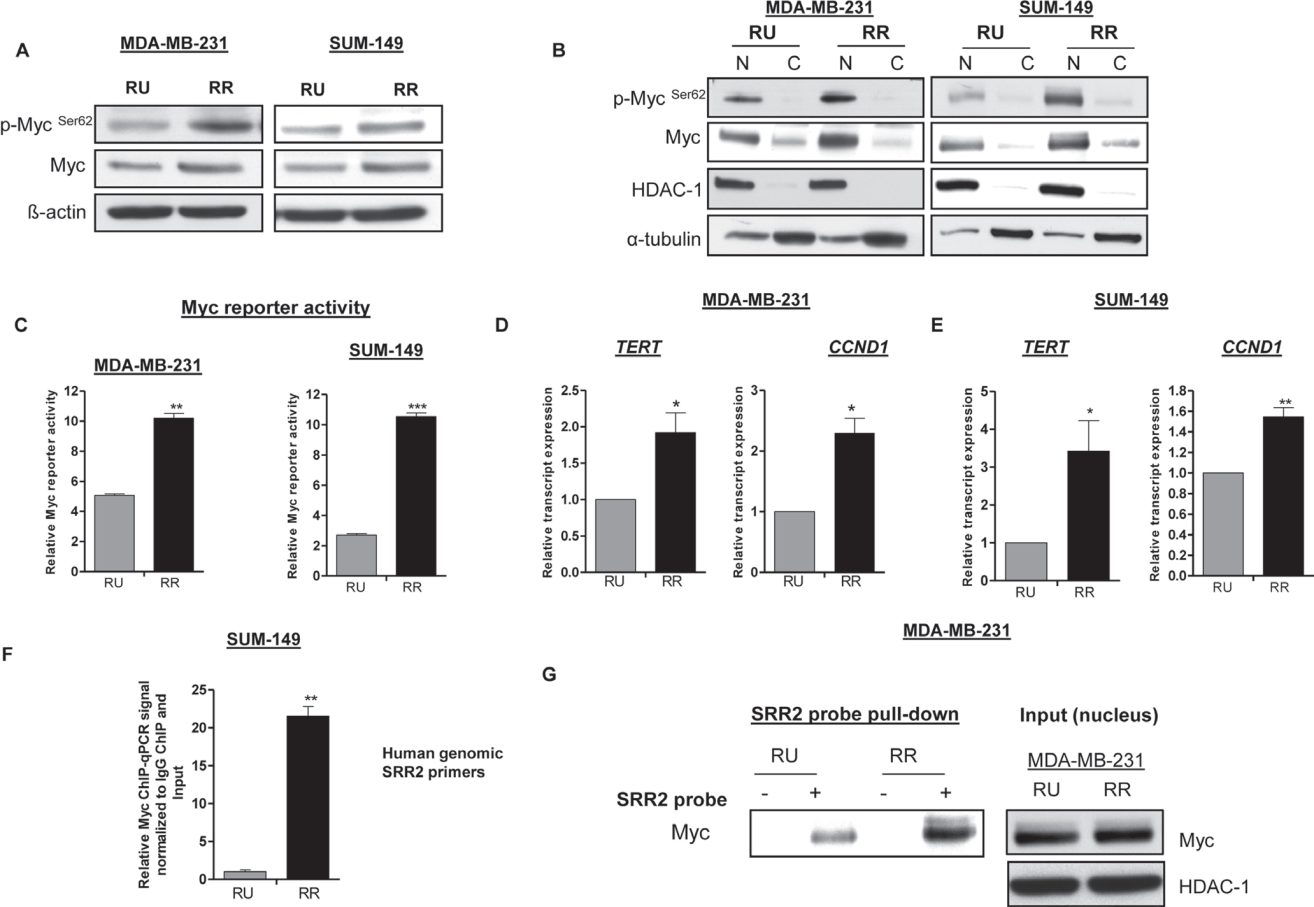
RR cells exhibit higher level of Myc and transcription activity compared to RU cells (**A**) Western blot was performed to assess p-Myc ^Ser62^ and Myc expression in RU and RR cells derived from MDA-MB-23 and SUM-149 cells. β-actin protein expression was used as a loading control of the experiment. (**B**) The subcellular localization of p-Myc^Ser62^ and Myc in RU and RR cells derived from MDA-MB-231and SUM-149 cells were assessed by the nuclear/cytoplasmic fractionation assay. HDAC-1 and α-tubulin were used as a loading control of the experiment. (**C**) Myc specific reporter activity was measured using the Cignal Myc Reporter Assay Kit in RU and RR cells derived from MDA-MB-231and SUM-149 cells. (**D–E**) The relative mRNA levels of Myc transcript targets, *TERT* and *CCND1* were measured by qRT-PCR in RU and RR derived from MDA-MB-231and SUM-149 cells. The mRNA expression levels were normalized with GAPDH. (**F**) Chromatin-immunoprecipitation-qPCR (ChIP-qPCR) was performed in RU and RR cells derived from SUM-149 cells to determine the Myc binding to SRR2 DNA sequence. (**G**) Immunoprecipitation assay was performed using SRR2 probe to determine Myc binding in RU and RR cells derived from MDA-MB-231 cells. Western blot in the right panel showed the input of the pull-down assay. HDAC-1 was used as a loading control of the input of the experiment.

We then assessed the protein expression level of Myc in purified RU and RR cells. As shown in Figure 1A, we found that RR cells derived from both MDA-MB-231 and SUM-149 cells expressed a higher level of Myc as compared to RU cells. We also evaluated the expression of the phosphorylated form of Myc, p-Myc^Ser62^, the functionally active form of Myc [14], which was found to be substantially higher in RR cells in both cell lines (Figure 1A). Using nuclear/cytoplasmic fractionation, we found that Myc in both RU and RR cells was mainly localized to the nuclei in both cell lines and was found to be higher in RR cells in both cell lines (Figure 1B). The same pattern was found for p-Myc^Ser62^ (Figure 1B).

To further support the relevance of Myc in RU and RR cells, we measured Myc-specific reporter activity using the Cignal Myc Reporter Assay Kit. As shown in Figure 1C, we found that RR cells derived from both MDA-MB-231 and SUM-149 cell lines expressed significantly higher Myc reporter activity as compared to their RU counterparts. Furthermore, we evaluated the gene expression levels of two known Myc gene targets, *TERT* and *CCND1* [21], and these genes expression were found significantly higher in RR cells derived from both cell lines (Figure 1D–1E). Furthermore, we performed chromatin immunoprecipitation-qPCR (ChIP-qPCR) to assess the interactions between Myc and the genomic SRR2 sequences, and we found that Myc binding to the genomic SRR2 sequences was significantly more frequent in RR cells than RU cells derived from SUM-149 cells (Figure 1F). Lastly, we performed immunoprecipitation experiments using a biotinylated SRR2 probe and found that the SRR2-Myc binding was substantially higher in RR cells derived from MDA-MB-231 cells (Figure 1G).

### Myc is a key regulator of the differential SRR2 reporter activity in TNBC cells

**Figure 2:**
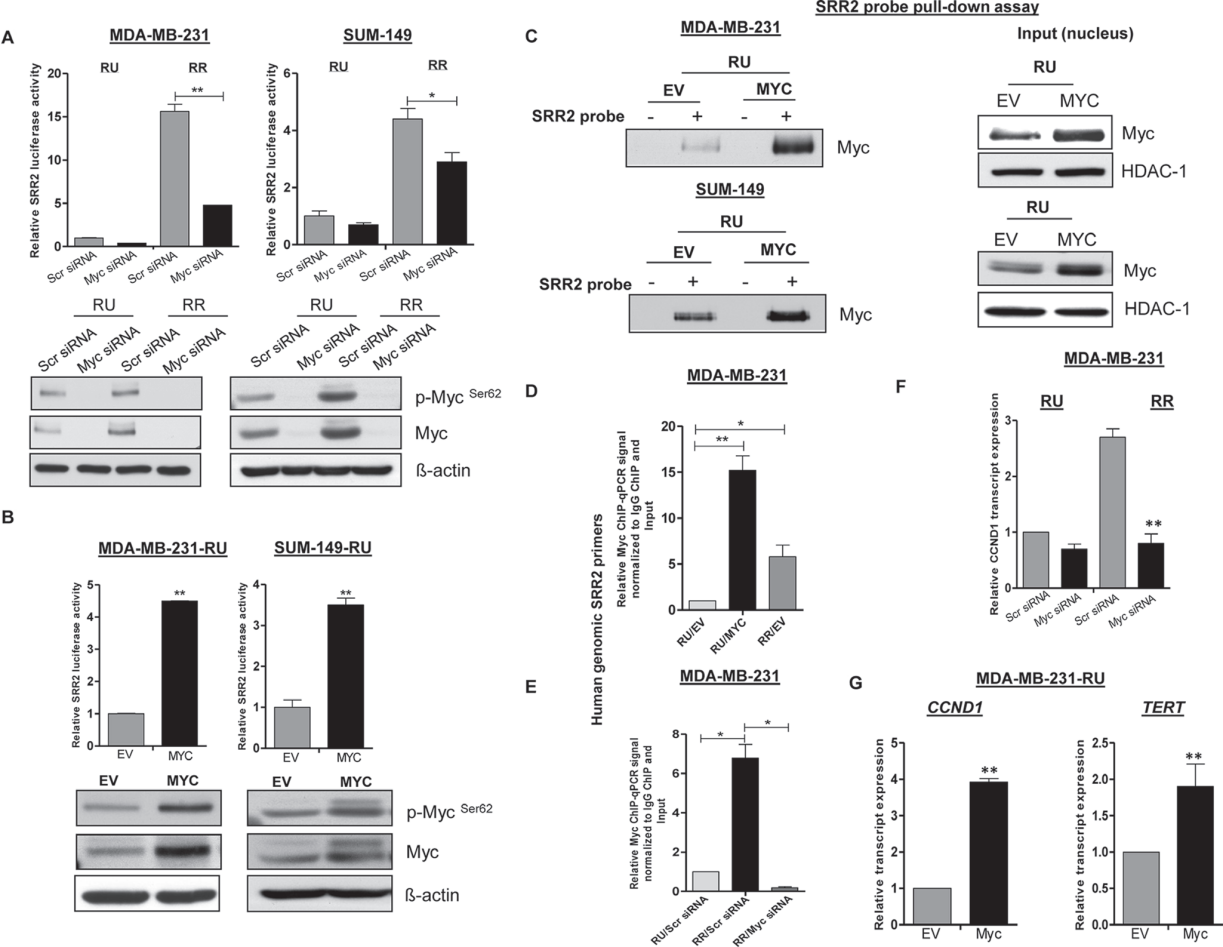
Differential Myc expression contributes to differential SRR2 reporter activity and differential binding capacity (**A**) The impact of Myc siRNA knockdown on the SRR2 reporter activity was measured by luciferase activity in RU and RR cells derived from MDA-MB-231 and SUM-149 cells. The western blots below showed the Myc knockdown efficiency. (**B**) RU cells derived from both TNBC cell lines were transiently transfected with pcDNA3.3-Myc. pcDNA empty vector (EV) was included as a negative control. The impact of enforced transient transfection of *MYC* on the SRR2 reporter activity was measured by luciferase activity. The western blots below showed the *MYC* transfection efficiency. β-actin protein expression was used as a loading control of the experiment. (**C**) RU cells derived from both TNBC cell lines were stably transfected with *MYC* and EV (negative control). The SRR2 probe pull-down assay was performed to assess the Myc and SRR2 binding in RU-*MYC* and RU-EV cells. The western blots in the right panel showed the input of the pull-down assay. HDAC-1 was used as a loading control of the experiment. (**D**) ChIP-qPCR was performed in RU-*MYC* and RU-EV cells derived from MDA-MB-231 cells to determine the Myc binding to SRR2 DNA sequence. RR-EV cells were included as a control of the experiment. (**E**) The impact of Myc siRNA knockdown on the Myc binding to SRR2 DNA sequence was performed using ChIP-qPCR in RR cells derived from MDA-MB-231 cells. RU cells transfected with scrambled siRNA were included as a control of the experiment. (**F**) The relative mRNA level of *CCND1* was measured by qRT-PCR after Myc knockdown in RU and RR cells derived from MDA-MB-231 cells. The mRNA expression level was normalized with GAPDH. (**G**) The relative mRNA level of Myc transcript targets, *CCND1* and *TERT* was measured by qRT-PCR in RU-*MYC* and RU-EV cells derived from MDA-MB-231 cells. The mRNA expression levels were normalized with GAPDH.

To provide additional support to the concept that the Myc expression level is an important regulator of the SRR2 reporter activity in TNBC cells, we down-regulated Myc expression using siRNA. As shown in Figure 2A, the SRR2 reporter activity in RR cells was reduced by 40–60% in MDA-MB-231 and SUM-149 cells; no appreciable effect was observed in RU cells when they were treated with Myc siRNA. The efficiency of Myc knockdown is illustrated in the lower panel of Figure 2A. In comparison, when we enforced Myc expression by Myc plasmid transient transfection in RU cells derived from both TNBC cell lines, we found a 4-fold increase in the SRR2 reporter activity (Figure 2B). Transfection efficiency of this experiment is illustrated in the lower panel of Figure 2B. Next, we generated stable transfected Myc cells, derived from RU cells from MDA-MB-231 and SUM-149 cells. These cells were labeled RU-EV (empty vector) and RU-*MYC*. Similar to the transient transfection experiment, the SRR2 reporter activity increased by approximately 4-fold in *MYC*-overexpressed stable cell clones compared to those transfected with the empty vector (EV) plasmid in both cell lines (data not shown).

To explore if the Myc expression level regulates SRR2 activity through direct protein-DNA interaction, we performed immunoprecipitation and ChIP-qPCR experiments. As shown in Figure 2C, RU-*MYC* cells from both cell lines resulted in substantially more Myc protein immunoprecipitated with SRR2 probe as compared to RU-EV cells. Similarly, in ChIP-qPCR, Myc bound significantly more frequently to genomic SRR2 DNA sequence in RU-*MYC* cells (approximately 15-fold higher) as compared to RU-EV cells derived from MDA-MB-231 cells (Figure 2D). On the contrary, siRNA knockdown of Myc in RR cells abrogated the binding of Myc with genomic SRR2 sequences (Figure 2E).

Correlating with these findings, upon Myc siRNA knockdown, we observed a significant decrease in the mRNA level of a known Myc downstream target, *CCND1* [21], in the MDA-MB-231 RR cells (Figure 2F); no appreciable effect was observed in RU cells. Furthermore, the mRNA level of *CCND1* and *TERT*, were significantly up-regulated in RU*-MYC* cells derived from MDA-MB-231 cells (Figure 2G). Taken together, our collected data strongly suggests that differential Myc expression in TNBC cells is a crucial factor dictating the observed heterogeneous SRR2 reporter activity, SRR2-Myc binding and expression of Myc downstream targets.

### Differential Myc expression underlies distinct cancer stem-like features within heterogeneous TNBC cell lines

**Figure 3:**
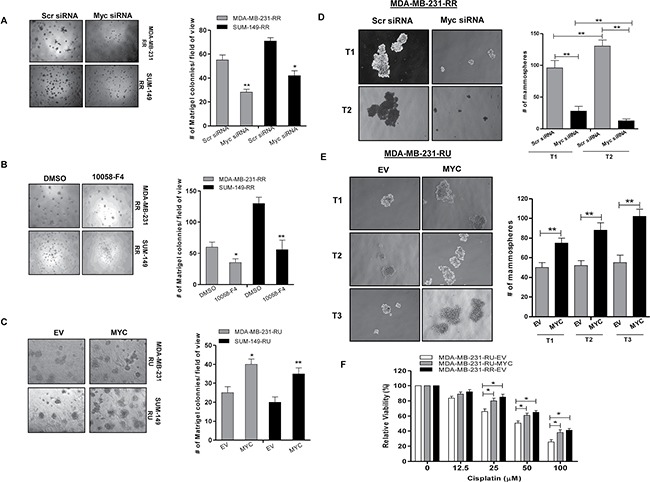
Myc expression contributes to cancer stem-like features in TNBC cell subsets Matrigel colony formation assay was performed after (**A**) Myc knockdown by siRNA in RR cells derived from MDA-MB-231 and SUM-149 cells and (**B**) Myc inhibition using 10058-F4 (10 μM) treatment in RR cells derived from MDA-MB-231 and SUM-149 cells. 2500 cells/well were seeded into the 8 well chambers. Colonies were counted and images were taken on Day 7. (**C**) Matrigel colony formation assay was performed to compare the colony formation ability in RU-*MYC* and RU-EV cells derived from MDA-MB-231 and SUM-149 cells. 2500 cells/well were seeded into the 8 well chambers. Colonies were counted and images were taken on Day 7. (**D**) Serial Mammosphere formation assay was performed to compare the mammosphere formation ability after Myc knockdown in RR cells and (**E**) RU-*MYC* and RU-EV cells, derived from MDA-MB-231 cells. Mammospheres were counted and images were taken on Day 7 for each passage. (**F**) MTS assay was performed to determine the cisplatin resistance in RU-*MYC* and RU-EV cells derived from MDA-MB-231 cells. RR-EV cells were included as a control of the experiment.

**Figure 4:**
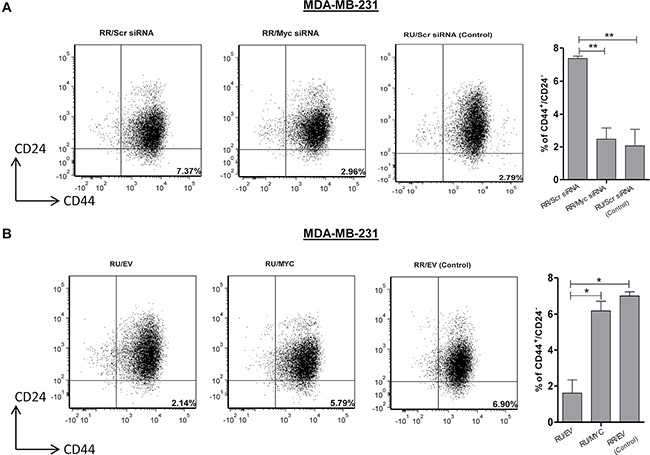
Myc expression regulates CD44^+^/CD24^−^ cell population in TNBC cell subsets (**A**) Flow cytometry was performed to measure CD44^+^/CD24^−^ cell population after Myc knockdown by siRNA in RR cells derived from MDA-MB-231 cells. Bar diagram represents the summary of 3 independent experiments. RU cells transfected with scrambled siRNA were included as a control of the experiment. (**B**) CD44^+^/CD24^−^ cell population was measured using Flow cytometry in RU-*MYC* and RU-EV cells derived from MDA-MB-231 cells. Bar diagram represents the summary of 3 independent experiments. RR-EV cells were included as a control of the experiment.

To evaluate the relevance of Myc in the context of cancer stem-like features within TNBC cells we first performed the Matrigel colony formation assay. As shown in Figure 3A–3B, RR cells treated with either Myc siRNA or 10058-F4 (a pharmacological inhibitor of Myc which inhibits Myc-Max heterodimerization [22]) formed a significantly lower number of Matrigel colonies compared to their negative controls in both TNBC cell lines. In comparison, RU-*MYC* cells derived from both TNBC cell lines formed a significantly higher number of Matrigel colonies compared to RU-EV cells (Figure 3C). Secondly, we utilized the serial mammosphere assay, which is a method used to measure cancer stemness [23]. As shown in Figure 3D, RR cells treated with Myc siRNA formed significantly fewer primary (T1) and secondary mammospheres (T2) as compared to the cells treated with the negative control siRNA. Further passage (i.e. T3) was not possible since RR cells treated with Myc siRNA did not yield sufficient number of T2 cells. In comparison, RU-*MYC* cells derived from MDA-MB-231 cells formed significantly more mammospheres compared to RU-EV cells at T1, T2 and T3 mammosphere (Figure 3E). Thirdly, we found that RU-*MYC* cells derived from MDA-MB-231 cells exhibited significantly higher cisplatin resistance compared to RU-EV cells (Figure 3F). Lastly, as CD44^+^ and CD24^−^ cells are well-known stem cell markers in breast cancer [4], we quantified the frequency of the CD44^+^/CD24^−^ cell population within TNBC cell lines. As shown in Figure 4A, siRNA knockdown of Myc in RR cells significantly decreased the size of the CD44^+^/CD24^−^ cell population, from 7% to 2%. In comparison, RU-*MYC* cells showed a significantly increase in the size of the CD44^+^/CD24^−^ cell population compared to RU-EV cells, from 1.5% to 6% (Figure 4B).

### MAPK/ERK signaling regulates SRR2 activity via Myc

**Figure 5:**
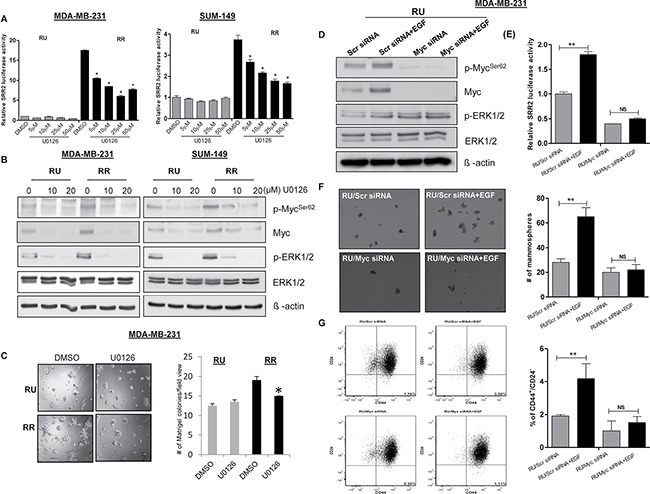
MAPK/ERK pathway regulates SRR2 reporter activity via Myc and regulates cancer stem-like features in TNBC cell subsets (**A**) The SRR2 reporter activity was measured in RU and RR cells derived from MDA-MB231 and SUM-149 cells after MEK inhibitor (U0126) treatment. (**B**) Western blot was performed to assess p-Myc^Ser62^, Myc, pERK1/2 and ERK1/2 expression in U0126 treated RU and RR cells derived from MDA-MB-231 and SUM-149 cells. β-actin protein expression was used as a loading control of the experiment. (**C**) Matrigel colony formation assay was performed to measure colony formation ability after U0126 treatment (10 μM) in RU and RR cells derived from MDA-MB-231 cells. 2500 cells/well were seeded into the 8 well chambers. Colonies were counted and images were taken on Day 7. (**D**) Western blot was performed to assess the protein expression of p-Myc^Ser62^, Myc, pERK1/2 and ERK1/2 after Myc knockdown followed by EGF (10 ng) treatment in RU cells derived from MDA-MB-231 cells. β-actin protein expression was used as a loading control of the experiment. (**E**) The SRR2 reporter activity was measured after Myc knockdown followed by EGF treatment (10 ng) in RU cells derived from MDA-MB-231 cells. (**F**) Mammosphere assay was used to evaluate mammosphere formation ability after Myc knockdown followed by EGF treatment (10 ng) in RU cells derived from MDA-MB-23 cells. Mammospheres were counted and images were taken on Day 7. One representative image has been shown here. (**G**) CD44^+^/CD24^−^ cell population was measured using Flow cytometry after Myc knockdown followed by EGF treatment (10 ng) in RU cells derived from MDA-MB-231 cells. Bar diagram represents the summary of 3 independent experiments.

Myc is known to be a downstream target of multiple signal transduction pathways, including the Wnt canonical pathway, JAK/STAT and RAS/RAF/MAPK pathways [24]. We did not observe differential expression of active β-catenin, p-AKT and p-STAT3 in RU and RR cells (data not shown). It has been previously shown that the MAPK/ERK pathway can up-regulate Myc expression in ER+ breast cancer [16]. Thus, we asked if this pathway also regulates the expression of Myc, the RU/RR phenotype, and thus the differential Myc-induced tumorigenic properties in TNBC. As shown in Figure 5A, U0126, a pharmacologic inhibitor of MAPK kinase, induced a significant and dose-dependent decrease in the SRR2 reporter activity in RR cells derived from both TNBC cell lines; no appreciable changes were found in RU cells upon U0126 treatment in both TNBC cell lines (Figure 5A). By western blots, we found that RR cells show substantially higher p-ERK1/2 expression as compared to RU cells derived from both MDA-MB-231 and SUM-149 cell lines (Figure 5B). We also confirmed that U0126 treatment led to a substantial reduction in p-ERK1/2, Myc and p-Myc^Ser62^ expression in both RU and RR cells derived from both TNBC cell lines (Figure 5B). Furthermore, U0126-induced reduction in Myc protein expression resulted in a significant decrease in Matrigel colony formation ability in RR cells (Figure 5C) derived from MDA-MB-231 cells; no significant changes were found in RU cells in the same experiment. We repeated the same experiment using SUM-149 cells and observed similar results (Supplementary Figure 2A–2B).

To further support our hypothesis that the MAPK/ERK/Myc axis is a key factor for the RU/RR phenotypic dichotomy, we treated RU cells with epidermal growth factor (EGF), which is known to activate the MAPK/ERK signaling pathway [25]. We found that EGF treatment increases the expression of Myc, SRR2 reporter activity and size of Matrigel colonies (Supplementary Figure 3A-3C). Further, we found that RU cells have a higher CD44^+^/CD24^−^ cell population upon EGF treatment compared to the untreated cells (Supplementary Figure 3D). To further validate that EGF-treated upregulation is dependent on Myc expression in RU cells, we down-regulated Myc expression using siRNA treatment followed by the EGF treatment. As shown in Figure 5D–5G, Myc siRNA treatment substantially decreased the biological effects of EGF in RU cells.

### Myc co-localizes with CD44 in a cell subset in primary patient tumors

**Figure 6:**
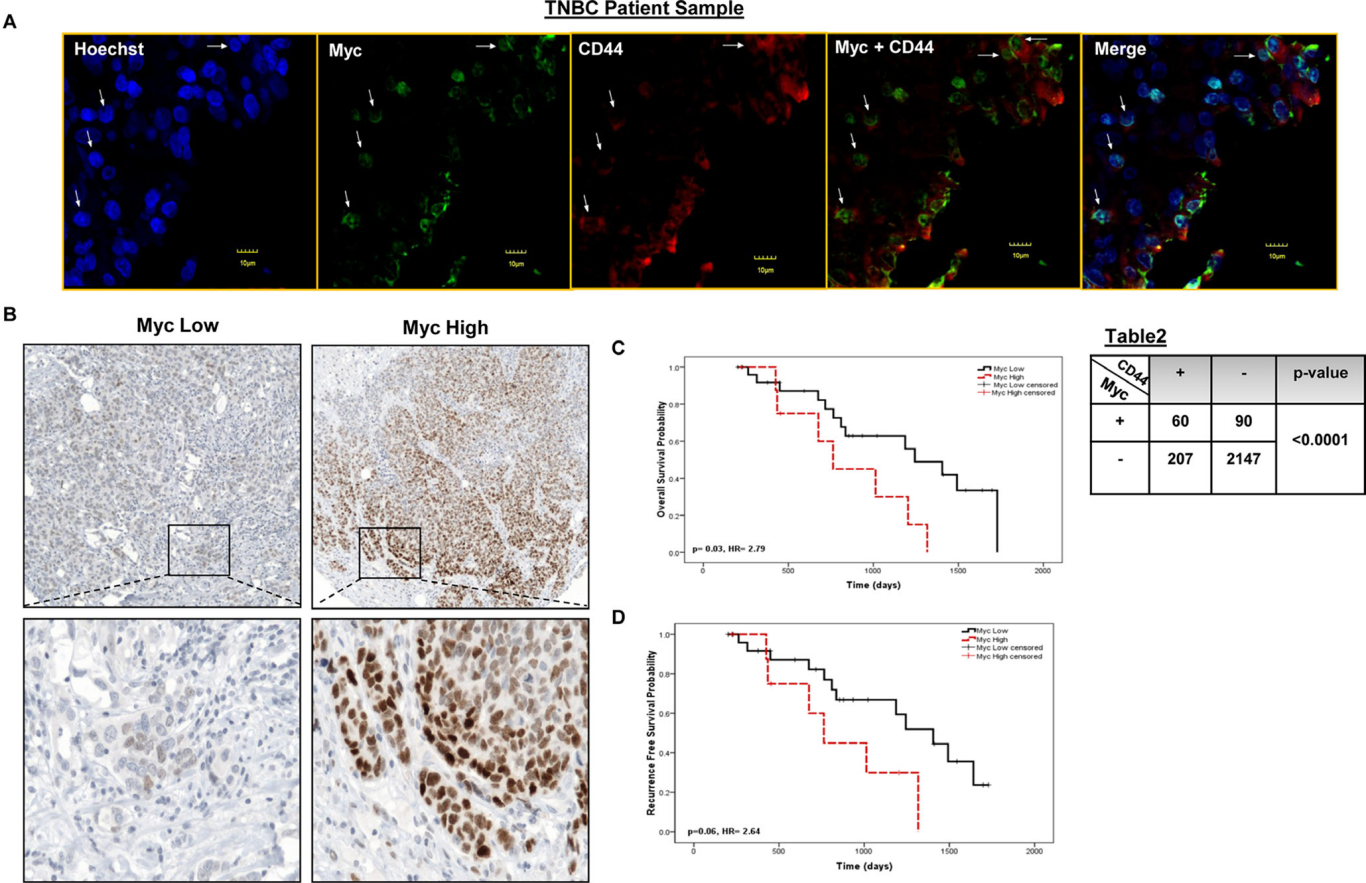
Myc co-localizes with CD44 in distinct tumor sub-populations and has prognostics significance in primary TNBC tumors (**A**) Immunofluorescence staining was performed in 5 primary TNBC tumors for Myc and CD44. As shown in the lower right panel, Myc (nucleus) was significantly (*p* < 0.0001) co-localized with CD44 (membrane) in patient tumor samples (*Fisher's exact test*). Four-five random fields of 5 patient tumor samples were chosen under the microscope (Χ400). One representative image has been shown here. (**B**) Immunohistochemistry was performed to evaluate Myc expression in a cohort of 35 TNBC patient tumors. Kaplan-Meier survival curves depicting (**C**) five years overall survival (HR = 2.79, 95% CI: 1.05–7.42) and (**D**) recurrence free survival (HR 2.64, 95% CI: 0.93–7.5) outcome of Myc expression in TNBC patient tumors.

Our *in vitro* data strongly suggests that RR cells are enriched with cells expressing CD44, a marker of cancer stem cells in TNBC [4]. To verify this phenomenon in primary TNBC patient tumors, we performed double immunofluorescence (Myc and CD44) and confocal microscopy in 5 primary TNBC patient tumors. As illustrated in Figure 6A, we were able to identify 4 different cell populations: cells double positive for both markers, cells singly positive for Myc or CD44, and cells negative for both markers. Three to five random fields were examined per case, and approximately 2000 cells were counted in total for each case. As summarized in the lower right panel, we found a significant correlation (*p* < 0.0001, *Fisher's exact* test) between the Myc and CD44 co-localization within the primary TNBC tumor samples. Taken together, our collected data strongly suggests that the co-localization of Myc and CD44 is not a cell line-specific phenomenon and Myc-expressing cells are enriched with cancer stem cells.

### High proportion of Myc-expressing cells correlates with a worse clinical outcome

In order to assess the clinical significance of Myc in TNBC, we correlated its expression with the overall survival (OS) and recurrence free survival (RFS) in cohort of TNBC patients (*n* = 35). Representative Myc staining, with one case showing low Myc expression and one case showing high Myc expression, are shown in Figure 6B (400Χ). Using the receiver operator characteristics (ROC) curves, we estimated that the cut-off point for Myc was 26.7; thus, tumors with a Myc score of ≤ 26.7 were assessed Myc low and those with a Myc score of > 26.7 were assessed Myc high. Of the 35 cases, 26 tumors were Myc low and 9 tumors were Myc high. As shown in Figure 6C, patients with Myc high had a significantly lower OS with a HR 2.79 (*p* = 0.03, 95%, CI: 1.05–7.42). We also found that patients with Myc high tumors had a shorter RFS, with a HR 2.64 (*p* = 0.06, 95% CI: 0.93–7.5) than those with Myc low tumors, although the difference is just short of statistical significance (Figure 6D).

## DISCUSSION

We have previously identified a novel intra-tumoral heterogeneity in TNBC cell lines and primary patient tumors based on the differential responsiveness to the SRR2 reporter [9]. The observed RU/RR dichotomy appears to be a widespread phenomenon, as we have made similar observations in a number of other cancer types, including ER+ breast cancer [10], esophageal cancer [26] and ALK-positive anaplastic large cell lymphoma [27]. Importantly, in all of these 3 models, the RR phenotype is also associated with significantly more cancer stemness. Similar to our studies, Iglesias *et al*., [28] also employed the SRR2 reporter and found intra-tumoral heterogeneity in ER+ breast cancer; importantly, this small subset of cells with SRR2 responsiveness also displayed relatively high tumorigenicity. In the ER+ breast cancer and ALK-positive anaplastic large cell lymphoma models, Sox2 was found to be the key determinant of the SRR2 reporter activity (i.e. the RR phenotype), since siRNA knockdown of Sox2 in these cells resulted in a significant loss of cancer stemness features. In contrast, we found that Sox2 is not a key factor in regulating SRR2 responsiveness in TNBC, as Sox2 is lowly expressed in TNBC and siRNA knockdown of Sox2 did not result in any significant biological changes and SRR2 responsiveness [9]. Thus, one of the main objectives of the current study is to identify the regulator(s) of the RR phenotype and its associated cancer stemness features in TNBC.

One of the main findings of this study is that Myc, a transcription factor which has been implicated in cancer stemness and pluripotent properties [29, 30], is the key regulator of the RU/RR dichotomy in TNBC. First, Myc was found to be highly expressed and highly transcriptionally activated in RR cells as compared to RU cells. Second, through a series of siRNA knockdown and gene transfection experiments, Myc was found to significantly modulate the RU/RR phenotype and associated cancer stem-like features, including the proportion of the CD44^+^/CD24^−^ cell population, mammosphere formation ability, Matrigel colony formation ability and chemoresistance. Our collected data suggested that the expression level and transcription activity of Myc is the key determinant of the RR and cancer stem-like phenotypes in TNBC. To our knowledge, heterogeneous Myc expression and differential Myc transcriptional activity within breast cells have not been previously characterized or studied in detail.

Myc, an important transcription factor, regulates the expression of many genes involved in cellular metabolism, growth and proliferation [21]. It plays an important role in tumorigenesis and its deregulation has been found in a variety of cancers including TNBC [18, 21, 31, 32]. In breast cancer, Myc deregulation has been associated with poor outcome [18, 33, 34]. Recent studies have shown that Myc regulates cancer stemness features, including the tumor initiating ability [19], chemoresistance [35] and self-renewal ability [36]. Recently, Zhao *et al*., [36] showed that Myc regulates sphere formation ability and drives cancer-initiating stem cells in TNBC. In our study, we found that the differential expression of Myc in RU/RR cells regulates cancer stem-like features (size of the CD44^+^/CD24^−^ cell population, resistance to cisplatin and serial mammosphere ability) in TNBC. Our immunofluorescence/confocal microscopy results in primary TNBC samples again have provided further evidence to support that high Myc expression correlates with cancer stemness in TNBC. In keeping with this concept, the Myc expression level has been reported to be relatively high in CSCs derived from several cancer types as compared to the bulk cell population [37, 38]. This observation correlates well with our findings as we also found that the RR cell subset (more tumorigenic and cancer stem-like features) derived from TNBC cells have relatively high Myc expression as compared to the general cell pool (RU) cells. Taken together, we report that Myc expression is heterogeneous within TNBC tumors and cell lines, resulting in differential observed Myc downstream gene regulation and differential observed tumorigenic phenotypes within tumors.

We have investigated the mechanisms that might have contributed to the significant difference in Myc expression between RU and RR cells. Myc is a downstream target of multiple signal transduction pathways, including the Wnt canonical pathway, RAS/RAF/MAPK and JAK/STAT pathways [24]. Except for p-ERK1/2 expression, we did not observe differential expression of active β-catenin, p-AKT and p-STAT3 (data not shown) in RU and RR cells. Indeed, experimental stimulation of the MAPK/ERK pathway (such as addition of EGF) significantly up-regulated Myc expression, SRR2 reporter activity and the associated stem-like features in RU cells. Moreover, pharmacologic inhibition (U0126 inhibitor) of this signaling led to the opposite effects on RR cells derived from both MDA-MB-231 and SUM-149 cell lines. Similar to our findings, Tsai *et al*., [15], also reported that the MAPK/ERK pathway is involved in Myc stabilization in melanoma cancer cells as U0126 treatment abolished Myc and p-Myc^Ser62^ expression. Recently, Luo *et al*., [39] reported that ERK pathway drives and regulates stem-like cells expansion and tumorigenicity in breast cancer and Horst *et al*. [40] also reported that the MAPK signaling regulates intra-tumoral heterogeneity and contributes to stem-like features in colon cancer. To the best of our knowledge, there is no literature available regarding the MAPK/ERK/Myc axis regulated intra-tumor heterogeneity in TNBC. We believe that these data are complimentary to what has been published in the literature regarding the role of the MAPK/ERK pathway and cancer stemness.

Myc amplification and its correlation with clinicopathological characteristics in breast cancer are less consistent. Deming *et al*., [32] conducted a meta-analysis of 29 studies and found that Myc amplification exhibited significant association with risk of relapse and death (RR = 2.05, RR = 1.74). Further studies also have confirmed the correlation between Myc amplification and prognosis [33, 41–43]. In contrast, others have reported no association between Myc and clinicopathological characteristics [44, 45]. This could be largely due to breast tumor heterogeneity and diversity of research methodologies. Few studies have shown that Myc amplification occurs more frequently in TNBC [18, 46]. Similar to these studies, we also found that Myc expression was higher in TNBC and it had strong and significant association with short overall survival (HR = 2.79, *p* = 0.03), indicating that Myc may be used as a prognostic marker in TNBC. We also found an association (HR = 2.64) between short recurrence free survival and high Myc expression, however this association was not statistically significant (*p* = 0.06) and the explanation can be the small sample size (*n* = 35). We found that out of 29 ER+ breast cancer cases, only 1 case had higher Myc expression (data not shown), and this finding indicates that Myc expression is limited to TNBC. We recognize that the sample size of this study is relatively small, and further studies involving larger cohort are warranted.

In conclusion, we have identified that Myc is a key regulator of the RU/RR dichotomy in TNBC, which is in turn linked to stem-like features in these tumors. While the mechanisms responsible for the high level of Myc in RR cells is likely to be multi-factorial, we have identified the MAPK/ERK to be an important contributor, and the MAPK/ERK/Myc axis may serve as a therapeutic target. Our clinicopathologic studies suggest that Myc expression detectable by immunohistochemical also may hold prognostication value.

## MATERIALS AND METHODS

### Patient information and tumor microarray analyses

Pre-treatment tumor samples were accessed from the Alberta Cancer Research Biobank/CBCF Tumor Bank. Patient information was collected under research ethics board approval (HREB Biomedical). All patients received guideline based standard therapy. Tissue microarray (TMA) was generated from formalin fixed tumor samples obtained for primary breast cancer patients as described in detailed previously [47]. Evaluation of histology slides from tissue adjacent to the frozen samples indicated that at least 70% of the cells present were invasive tumor cells. A total of 60 primary breast tumor samples were examined and only ER, PR and HER2 negative cases were included in the analysis. A detailed summary of the clinical characteristics of the TNBC patient tumors is described in Table 1.

**Table 1:** Clinical characteristics of TNBC patients

Clinical parameters	Total patient number (*n* = 35)	
	Patient number	Percentage
**Median age at diagnosis (age range)**	30–89	
< 54 years	17	49%
≥ 54 years	18	51%
**Tumor Size**		
T1	14	40%
T2	17	49%
T3	0	0%
T4	4	11%
**Lymph node metastasis**		
N0	17	49%
N1	18	51%
N2	0	0%
N3	0	0%
**Stage**		
I	9	26%
II	22	63%
III	4	11%
IV	0	0%
**Distant Metastasis**		
M0	35	100%
M1	0	0%
**Overall grade**		
Low	3	9%
High	32	91%

### Cell culture and reagents

MDA-MB-231 cells were purchased from ATCC and cultured in DMEM high glucose media (Life Technologies, Grand Island, NY, USA) supplemented with 10% fetal bovine serum (FBS). SUM-149 cells were obtained from Dr. Sandra E. Dunn (University of British Columbia) through collaboration and were cultured in F12 media (Life Technologies, Grand Island, NY, USA) supplemented with 5% FBS, 5 μg/mL insulin, 1 μg/mL hydrocortisone, and 10 mM Hepes. Cell lines were virally infected twice with mCMV or SRR2 reporter as described previously [9]. Both cell lines have been authenticated using short tandem repeat DNA profiling (from TCAG Genetic Analysis Facility, Toronto, CA). U0126 (#9903, Cell Signaling), 10058-F4 (#F3680, Sigma), epidermal growth factor (EGF) (#E9644, Sigma) and Cisplatin (Sigma) reagents were used following the manufacturer's instructions.

### Immunofluorescence

Myc antibody (Y69) was purchased from Abcam (Cambridge, MA) and CD44 antibody was purchased from Millipore (MA) for immunofluorescence study. Immunofluorescence study was performed as per the manufacture's guidelines. 1:100 dilution for Myc antibody and 1:50 dilution for CD44 antibody were used for the staining. Tissue sections were viewed using a laser confocal microscope (Zeiss LSM 710).

### Immunohistochemistry

Immunohistochemistry (IHC) was performed with Anti-Myc (1/50, EP121, 395R-15; Cell marquee, Rocklin, CA) antibody, following the procedure described previously [48]. The average of three samples was used to define the staining for each patient. The scoring of the immunostained TMA was performed in an outcome blinded fashion according to training and guidelines.

### SRR2 probe pull-down assay

A biotinylated SRR2 probes was synthesized by Integrated DNA Technologies, USA. SRR2 sequence is 5′-AAGAATTTCCCGGGCTCGGG CAGCCATTGTGAT GCATATAG GATTATTCACGTGGTAATG-3′ in which Myc consensus sequence is underlined. 300μg nuclear protein was incubated with 3 pmole of either with or without biotin-labeled SRR2 probe for 30 min at room temperature. Then 75 μl streptavidin beads were added and the samples were incubated overnight by rotation at 4°C. The next day beads were washed 3 times with cold PBS and protein was eluted at 100^°^C in 4 × protein loading dye and loaded on SDS-PAGE gels and then processed for western blotting.

### Short interfering RNA and western blotting

Myc siRNAs (SMARTpool: ON-TARGET plus Myc siRNA, Dharmacon, Fisher Scientific, ON, Canada) or scrambled (Scr) siRNAs (ON-TARGETplus Non-targeting Pool, #477C20, Dharmacon, Fischer Scientific, ON, Canada) at 40 pmol per reaction (20 nM final concentration) and 5 μL of Lipofectamine RNAiMAX (Life Technologies, Grand Island, NY, USA) were added to 0.5 mL of OptiMEM media (Life Technologies, Grand Island, NY, USA). 500,000 cells were seeded 24 hours prior to transfection in normal culture medium in a 6-well plate format and cells were incubated with siRNAs for 48 hours before harvesting. Western blotting was performed as described previously [9]. Antibodies were all diluted in TBST supplemented with 5% BSA. Antibodies for phospho Myc Ser62 (E1J4K), Myc (D84C12), Phospho-Erk Thr202/Tyr204, T-Erk were purchased from Cell Signaling Technology (Danvers, MA). The expression of β-actin served as the loading control for all western blots.

### Myc reporter assay

Myc reporter activity was measured using the Cignal Myc Reporter Assay Kit (#336841, CCS012L, SABiosciences, Qiagen) as per the manufacturer's protocol.

### JASPAR protein motif analysis

SRR2 sequence used was TAATTAATGCAGAG ACTCTAAAAGAATTTCCCGGGCTC GGGCAGCCA TTGTGATGCATATAGGATTATTCACGTGGTAATG. The JASPAR vertebrate core database was used for the reporter sequence matching. Analysis was performed using R-3.0.0, with reports generated using RStudio and Sweave. The sequence is not particularly GC biased or skewed with a nucleotide distribution so a uniform background (even nucleotide distribution) will be assumed for Motif scoring on both strands. The set of JASPAR vertebrate core set of transcription factors (Downloaded April 17, 2013) was applied to the reporter sequence on both strands with a *p-value* < 0.001.

pcDNA3.3-Myc plasmid was a gift from Derrick Rossi (Addgene plasmid #26818) and pcDNA plasmid was purchased from Addgene (Cambridge, MA). Transient transfection of cells (500,000) with 3μg plasmid was performed using Lipofectamine 2000 (Life Technologies, Grand Island, NY, USA) and added to 0.5 mL of OptiMEM media (Life Technologies, Grand Island, NY, USA). Media was replaced with fresh media after 6 hours of transfection and cells were harvested after 48 hours of transfection. For stable cell lines 10 million RU and RR cells derived from MDA-MB-231 and SUM-149 cells were initially transfected with 15 μg pcDNA3.3-Myc and pcDNA plasmids and then cultured for 3–4 weeks in selection medium with increasing concentrations of G418 up to 400 ng/ml. Western blot study was performed to examine the stable cell lines.

### Nuclear-cytoplasmic fractionation

Nuclear and cytoplasmic proteins of cells were extracted using the NE-PER Protein Extraction Kit (#78833, Thermo Scientific, USA) according to the manufacturer's protocol. Histone deacetylase 1 (HDAC-1, Santa Cruz biotechnology Inc. USA) and α-tubulin were used as nuclear and cytoplasmic fraction markers for western blotting experiments.

### SRR2 luciferase reporter, flow cytometry and chromatin-immunoprecipitation q-PCR (ChIP-qPCR) assays

Luciferase reporter assay was performed using luciferase assay system kit (#E4530, Promega, Corporation, Madison, USA) according to the manufacturer's protocol, on costar white polystyrene opaque 96-well plates (#3912, Corning, NY, USA) and analyzed on the FLUOstar Omega multi-mode microplate reader (BMG Labtech, Ortenburg, Germany). Flow cytometry analysis was performed as described previously [9]. Chromatin immunoprecipitation (ChIP) was performed as described previously [11]. 5 μg of Myc antibody (sc-40, Santa Cruz) or 5 μg of mouse normal IgG antibody (sc-2025, Santa Cruz) were used. Human SRR2 primers; Forward primer: 5′-ACATTGTACTGGGAAGGGACA-3′, Reverse primer: 5′- GCAAGAACTGGCGAATGTG-3′ were used for qPCR.

### Matrigel, mammosphere formation and MTS assay

In the Matrigel assay, cells were seeded at 2500 cells/well in 200 μL of media atop of 40 μL of corning Matrigel matrix in 8-well slide chamber and pictures were taken on Day 7. U0126, EGF, or vehicle controls were added directly into media and incubated for the 7-days assay duration. In the mammosphere assay cells were seeded and cultured as previously described [9]. Briefly, cells were trypsinized and passed through a 40 μm cell strainer (BD, Franklin Lakes, New Jersey, USA) and seeded into ultra-low adherent plates (Corning, NY, USA) in MammoCult media (StemCell Technologies, Vancouver, BC, Canada) as per manufacturer's instructions. Mammosphere formed after 7 days were collected by centrifugation at 1000 rpm. For serial passaging, obtained mammosphere were dissociated into single cells and seeded again into ultra-low adherent plates (Corning, NY, USA). Cell viability was determined using the 3-(4,5-dimethylthiazol-2-yl)-5-(3-carboxymethoxyphenyl)-2-(4-sulfophenyl)-2H-tetrazolium, inner salt (MTS) assay (#G3580, Promega, Madison, WI, USA) according to the manufacturer's instructions.

### RNA extraction, cDNA synthesis, quantitative reverse transcription PCR (q-RT-PCR)

Total RNA extraction was performed with the Qiagen RNeasy Kit (Qiagen, Canada) according to the manufacturer's protocol. 1 μg of RNA was reverse transcribed using oligo-dT and Superscript II (Life Technologies, Grand Island, NY, USA) according to the manufacturer's protocol. 1 μL of the resulting cDNA mixture was added to the Platinum SYBR Green qPCR SuperMix-UDG with Rox (Life Technologies, Grand Island, NY, USA) and amplified with target gene-specific primers on the Applied Biosystems 7900HT (Carlsbad, CA; The Applied Genomics Centre, Edmonton, Alberta, Canada). All genes of interest were normalized to glyceraldehyde-3-phosphate dehydrogenase (GAPDH) transcript expression levels. Please see Supplementary Table 1 for list of primer sequences.

### Statistical analysis

The statistical analysis was performed by Graphpad Prism (La Jolla, CA) software. Significance of two independent groups of samples was determined by using Student's *t-test*. Statistical significance is denoted by *(*p* < 0.05), **(*p* < 0.01) and ***(*p* < 0.001). All graphs represent the average of at least 3 independent experiments with triplicates. Data are expressed as mean ± standard deviation (SD). Receiver operator characteristics (ROC) curve analysis was used as a measure of sensitivity versus specificity for determining the optimum cutpoint value for Myc expression. Overall survival (OS) and Recurrence free survival (RFS) was calculated by Kaplan-Meier analysis using SPSS (ver.17.0) software. Significant differences between Kaplan-Meier curves were measured by log-rank test. Hazards ratio (HR) and confidence interval (CI) are calculated as univariate test result using Cox regression model.

## SUPPLEMENTARY MATERIALS FIGURES AND TABLES


