# Supplementary material for: High Myc expression and transcription activity underlies intra-tumoral heterogeneity in triple-negative breast cancer
Source: Oncotarget. 2017 Mar 3;8(17):28101–15. doi: 10.18632/oncotarget.15891 (PMC5438634; doi:10.18632/oncotarget.15891)
Supplement: Supplementary file 1 [file oncotarget-08-28101-s001.pdf]

High Myc expression and transcription activity underlies intra-tumoral heterogeneity in triple-negative breast cancer

Supplementary Materials

A

	Name	p value	Sequence
1	USF1	0.0001	CACGTGA
2	ARNT	0.0002	CACGTG
3	E2F1	0.0001	TTTCCCGG
4	EVI1	0.0004	CATATAGGATTATT
5	RELA	0.0004	GGGAAATTCT
6	MYC	0.0002	TTCACGTGGT
7	NFκB	0.0002	TCACAATGG
8	FEV	0.0001	CGGGAAAT
9	NOBOX	0.0005	TAATTAAT
10	TFAP2A	0.0007	GCCCGAGCC
11	MAX	0.0009	TACCACGTGA
12	SPI1	0.0008	GGGAAAT
13	HOXA5	0.0003	CATTAATT
14	HIF1α	0.0004	CCACGTGA
15	TFCP2L1	0.0007	CATTGTGATGCATAT
16	SOX2	0.0008	CCATTGTGATGCATA

B

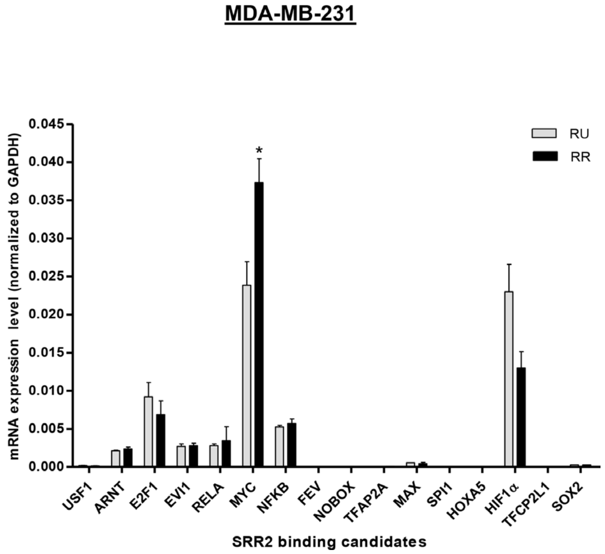

Supplementary Figure 1: Profiling the expression of putative SRR2-binding proteins. (**A**) SRR2 sequence harbour a number of unique JASPAR motif matches at *p*-value < 0.001. List of putative proteins with JASPAR motifs matching 81 bp SRR2 DNA sequence with *p*-value < 0.001. (**B**) RR cells exhibit higher Myc mRNA expression as compared to RU cells. Quantitative reverse transcriptase-PCR (qRT-PCR) was performed to measure the mRNA expression levels of the 16 SRR2 binding candidates in RU and RR cells derived from MDA-MB-231 cells. All the mRNA expression levels were normalized with GAPDH.

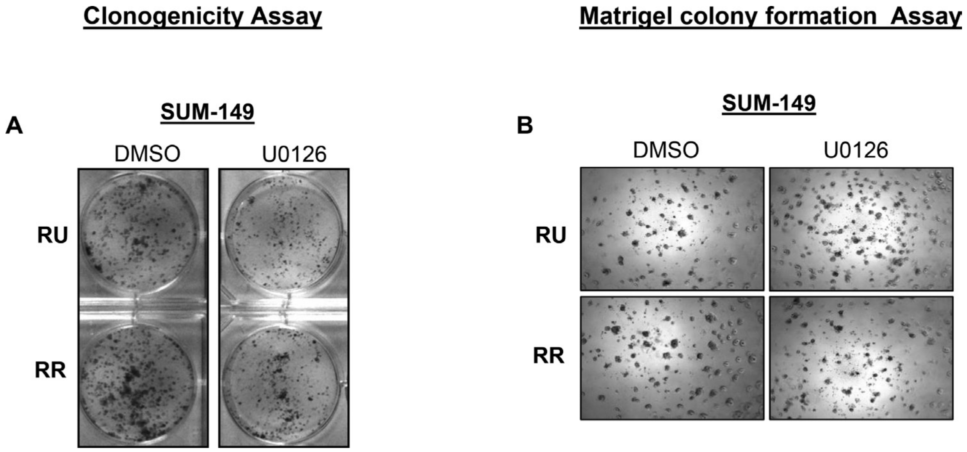

Supplementary Figure 2: MEK inhibitor U0126 inhibits clonogenicity and Matrigel colony formation ability and in RU and RR cells derived from SUM-149 cells. (**A**) 500 cells/well were seeded into the 12 well plate with 10 µM U0126 or DMSO and colonies were photographed on Day 15. (**B**) 2500 cells/well are seeded into the 8-well chamber Matrigel colony formation assay with 10 µM U0126 or DMSO and colonies were photographed on Day 7.

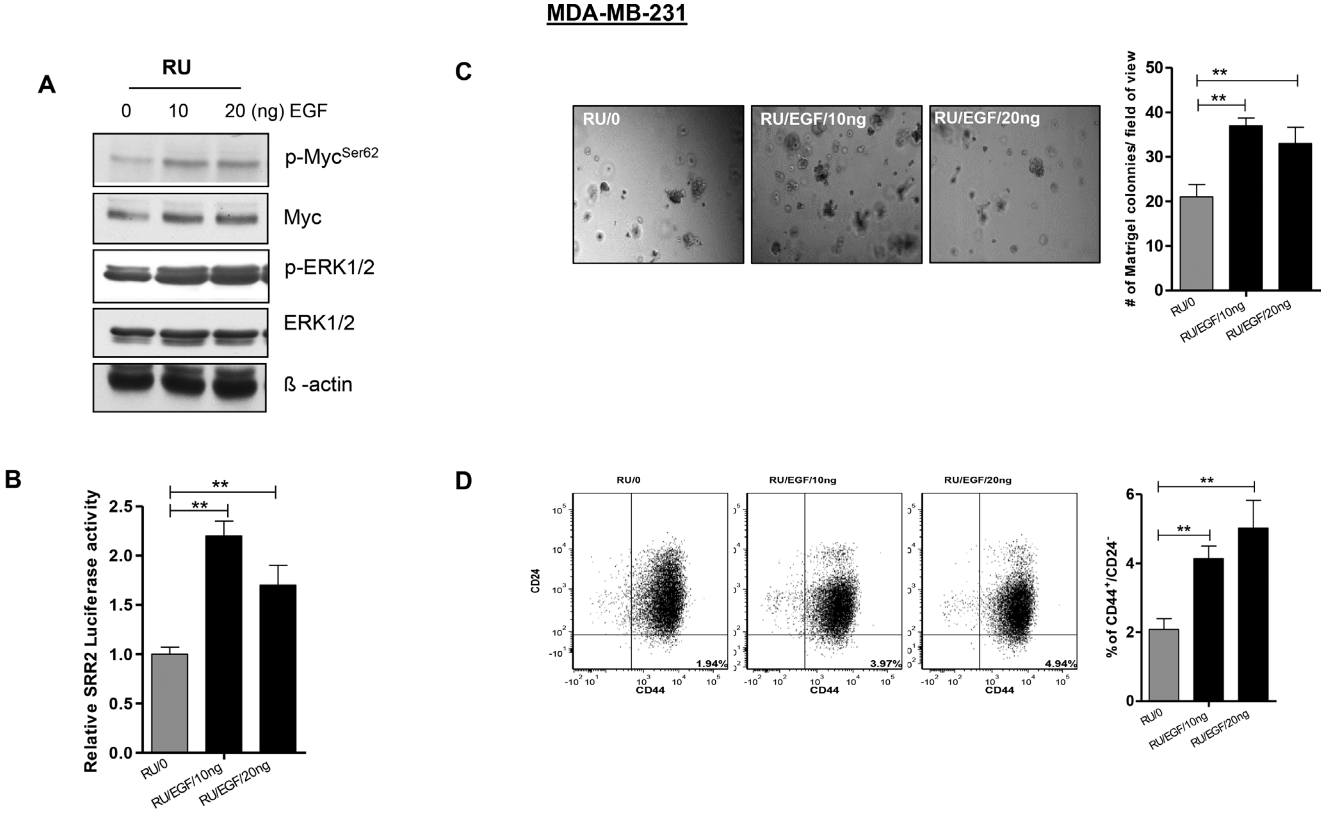

Supplementary Figure 3: EGF treatment induces SRR2 reporter activity and regulates stem-cell like features. RU cells derived from MDA-MB-231 cells were treated with EGF (10 and 20 ng/mL) for 24 hours. (**A**) Western blot was performed to assess Myc protein expression. β-actin protein expression was used as a loading control of the experiment. (**B**) SRR2 luciferase activity was measured after EGF treatment. (**C**) Matrigel colony formation assay was performed to assess colony formation ability after EGF treatment. One representative image has been shown here taken at day 7. (**D**) Flow cytometry was performed to measure $CD44^{+}/CD24^{-}$ cell population after EGF treatment.

Supplementary Table 1: List of primer sequences

Gene	Forward Primers	Reverse Primers
USF1	5′-CTGCTGTTGTTACTACCCAGG-3′	5′- TCTGACTTCGGGGAATAAGGG-3′
ARNT	5′-CTGCCAACCCCGAAATGACAT-3′	5′-CGCCGCTTAATAGCCCTCTG-3′
E2F1	5′- ACGCTATGAGACCTCACTGAA-3′	5′- TCCTGGGTCAACCCCTCAAG-3′
Evi1	5′-TATCCACGAAGAACGGCAATATC-3′	5′-CATGGAAACTTTTGGTGATCTGC-3′
RELA	5′-ATGTGGAGATCATTGAGCAGC-3′	5′-CCTGGTCCTGTGTAGCCATT-3′
MYC	5′-TACCCTCTCAACGACAGC AG-3′	5′-TCTTGACATTCTCCTCGGTG-3′
NFκB	5′-AACAGAGAGGATTTCGTTTCCG-3′	5′-TTTGACCTGAGGGTAAGACTTCT-3′
FEV	5′- CACGGCGAGTTCAAGCTCA-3′	5′- CTGGAAGTCGAAGCGGTAGG-3′
Nobox	5′-GAGACCCTCAAATCACCCCAA-3′	5′-GCCCCTTGTGAGTTCCCTTTT-3′
TFAP2A	5′-AGGTCAATCTCCCTACACGAG-3′	5′-GGAGTAAGGATCTTGCGACTGG-3′
MAX	5′-GAGAGCGACGAAGAGCAACC-3′	5′-GCACTTGACCTCGCCTTCT-3′
SPI1	5′-GTGCCCTATGACACGGATCTA-3′	5′-AGTCCCAGTAATGGTCGCTAT-3′
HOXA5	5′- AACTCATTTTGCGGTCGCTAT-3′	5′- TCCCTGAATTGCTCGCTCAC-3′
HIF1α	5′-GAACGTCGAAAAGAAAAGTCTCG-3′	5′-CCTTATCAAGATGCGAACTCACA-3′
TFCP2L1	5′-CAGCCCGAGCACTACAACC-3′	5′-CTCCCAGCTTCCGATTCTCC-3′
SOX2	5′- GCCGAGTGGAAACTTTTGTCG-3′	5′- GGCAGCGTGTACTTATCCTTCT-3′
GAPDH	5′- GGAGCGAGATCCCTCCAAAAT-3′	5′- GGCTGTTGTCATACTTCTCATGG-3′
